# Investigating the role of hyperexpressed HCN1 in inducing myocardial infarction through activation of the NF-κB signaling pathway

**DOI:** 10.1515/biol-2022-0967

**Published:** 2024-12-31

**Authors:** Xiao Liang, Jie Zhang, Ya Luo

**Affiliations:** Department of Cardiology, The Third Affiliated Hospital of Zunyi Medical University (The First People’s Hospital of Zunyi), No. 98 of Phoenix Road, Huichuan District, Zunyi, 563000, Guizhou, People’s Republic of China; Department of Cardiology, The Third Affiliated Hospital of Zunyi Medical University (The First People’s Hospital of Zunyi), Zunyi, 563000, Guizhou, People’s Republic of China

**Keywords:** HCN1, IKKβ, myocardial infarction, NF-κB, PDTC

## Abstract

We investigated the protective effect of the NF-κB inhibitor, pyrrolidine dithiocarbamate (PDTC) on cardiomyocyte injury induced by HCN1 channel overexpression, and explored the underlying mechanisms. An HCN1 overexpression vector was constructed and transfected into H9C2 cells, followed by PDTC treatment. The experiments comprised the following groups: control, control + PDTC, overexpression negative control, HCN1 overexpression (HCN1-OE), and combined HCN1-OE + PDTC groups. Cell proliferation was assessed using the CCK8 assay, while apoptosis and reactive oxygen species (ROS) levels were measured by flow cytometry. ELISA kits were used to determine the levels of malondialdehyde, superoxide dismutase, and interleukin-1 beta. The HCN1-OE group exhibited increased apoptosis, elevated ROS, and decreased survival. Western blot (WB) analysis revealed increased levels of p65, p-IκB, IKKβ, NLRP3, Beclin-1, and LC3 II/I proteins in the HCN1-OE group. PDTC treatment for 48 h post-HCN1-OE resulted in improved cell viability, reduced apoptosis, and decreased ROS in the HCN1-OE + PDTC group. Immunofluorescence and WB analysis indicated a reduction in HCN1 and NF-κB pathway protein levels in the HCN1-OE + PDTC group. In conclusion, PDTC provided protection against HCN1-induced cardiomyocyte injury, potentially by modulating inflammatory cytokines and regulating the IKKβ/IκB/NF-κB signaling pathway.

## 1 Introduction

Acute myocardial infarction (AMI) is a critical condition that can lead to sudden cardiac death. Previous studies indicate that the hospitalization rate due to AMI increased from 3.7 per 100,000 in 2001 to 8.1 per 100,000 in 2006, and to 15.8 per 100,000 in 2011 [1]. Despite advancements in percutaneous coronary intervention techniques, many patients with acute coronary syndrome still experience varying degrees of heart failure symptoms during hospitalization [2]. Moreover, since necrotic myocardial cells lack regenerative capabilities post-infarction, some patients may progress to chronic heart failure. Clinical manifestations of this condition include abnormal myocardial wall segment movement, ventricular aneurysm formation, and dysrhythmia [3].

A 2017 study analyzing over 50 million Chinese health insurance records revealed that coronary artery disease accounts for 52.5% of heart failure cases. The average hospitalization cost per patient exceeds RMB 30,000, and 40.5% of patients are readmitted more than three times [4]. The high cost of hospitalization and the continuously declining quality of life have become major social issues faced by patients with myocardial infarction.

Currently, there is a substantial gap in understanding the genetic factors and molecular mechanisms contributing to AMI. Environmental risk factors alone are insufficient to fully explain the risks associated with the onset of AMI and mortality. Both rare and low-frequency genetic alterations significantly influence AMI. Therefore, it is imperative to explore the pathophysiological basis of heart failure following AMI, identify the underlying molecular mechanisms, and implement targeted treatments at an early stage. This approach aims to maximize the reversal of ventricular remodeling, improve the quality of life of patients, and reduce readmission rates [4,5].

Hyperpolarization-activated and cyclic nucleotide-gated (HCN) channels, intrinsic to cardiac pacemaker cells, are critical for cardiac function [6,7]. The characterization of HCN channels began in the late 1970s, and since then, four HCN channel members have been cloned. However, channelopathies related to HCN require more comprehensive research [8]. The primary role of HCN channels in cardiac pacemaker cells is to initiate the spontaneous activities that regulate heart rate. Mutations or deletions in these channels can lead to inheritable cardiac dysrhythmic diseases [9–11].

The transcription factor NF-κB is a key regulator of immune and inflammatory responses. The mammalian NF-kappa B/Rel family consists of five proteins: p50, p52, p65 (Rel-A), c-Rel, and Rel-B [12]. These proteins can form either homogeneous or heterogeneous dimers and are bound by the inhibitory molecule IκB in resting cells to remain in an inactive complex [13]. Two different NF-κB signaling pathways have been identified: (1) the classic pathway, primarily activated by pathogens and inflammatory mediators and (2) the non-classic pathway, mainly triggered by developmental stimuli in lymphoid organs. The most common activation mechanism of NF-κB in the classical pathway involves the formation of p65:p50 heterodimers [14]. Excessive activation of P65, which leads to subsequent molecular transcription and activation, is a key factor in the pathogenesis of many chronic diseases, including rheumatoid arthritis, inflammatory bowel diseases, multiple sclerosis, and even neural degenerative diseases [13,14]. In cardiovascular disease, elevated NF-κB activity contributes to myocardial damage by inducing autophagy [15].

## 2 Materials and methods

### 2.1 Experimental materials and methods

The study utilized the following materials: DMEM (high glucose) complete medium (KGM12800S, KeyGenBiotec), DMEM incomplete (high glucose) medium (KGM12800N, KeyGenBiotec), trypsin-EDTA solution (T1300, Solarbio), 1× PBS (0.01 M, pH 7.4; KGB5001, KeyGenBiotec), CCK-8 cell proliferation detection kit (KGA317, KeyGenBiotec), Lipofectamine™ 3000 Transfection Reagent (Invitrogen™, L3000015), OPTI-MEM^®^ I reduced serum medium (31985-062, Gibco), FBS (10099-141, Gibco), and pyrrolidine dithiocarbamate (PDTC; HY-18738, MCE).

Additionally, reactive oxygen species (ROS) assay kit (KGT010-1 100 assays, KeyGenBiotec), annexin V-FITC/PI apoptosis kit (AP101-100-kit, MULTI SCIENCES), RIPA buffer (C1053, Beijing Applygen technologies Inc.), BCA protein assay kit (E-BC-K318-M,Wuhan Elabscience), marker (#26617, Thermo), PVDF membrane (IPVH00010, Millipore), defat dried milk for western blot (WB; P1622, Beijing Applygen Technologies Inc.), bovine serum albumin (BSA; A8020, Beijing Solarbio Life Sciences), ECL luminescent liquid (RJ239676, Thermo Fisher), 4% paraformaldehyde (P1110, Solarbio), and ready to use DAPI stain (KGA215-50, KeyGen Biotech) were employed.

Primary antibodies used included HCN1 (55222-1-ap, Proteintech, 1/200), and secondary antibodies included Cy3 goat anti-rabbit IgG(H + L) (AS007, ABclonal, 1/200). The malondialdehyde (MDA) kit (E-EL-0060c, Elabscience), superoxide dismutase (SOD) kit (A001-3-2, Nanjing Jiancheng Bioengineering Institute), BCA protein assay kit (E-BC-K318-M, Elabscience), NovoCyte™ flow cytometer (NovoCyte 2060R, Hangzhou ACEA Biosciences), automatic ELISA reader (SuPerMax 3100, Shanghai Shanpu), ultrasensitive chemiluminescence imaging system (ChemiDoc^TM^ XRS+, Shanghai Bio-Rad), and an automated CL imaging analysis system (Tanon-5200, Shanghai Tanon) were also used.

### 2.2 CCK8 detection

Cells were cultured until they reached 80% confluence, then digested and seeded in a 96-well plate at a density of 1 × 10^4^ cells per well. After cell adhesion, various concentrations of medications were administered. After 24 h, 100 μL of the existing medium in each well was replaced with fresh medium, and 10 μL of CCK8 reagent was added. The plates were incubated for 2 h and absorbance in each well was measured using an ELISA reader at a wavelength of 450 nm.

### 2.3 Flow cytometry detection

Approximately 1 × 10^6^ cells were collected and washed twice with PBS by centrifugation at 1,500 rpm for 3 min. The cells were resuspended in a 300 μL solution of pre-cooled 1× Annexin V-FITC binding buffer. A 5 μL solution of Annexin V-FITC and a 10 μL solution of PI were added to each well. After gentle mixing, the cells were incubated at room temperature in the dark for 10 min, and apoptosis rate was detected via flow cytometry.

For ROS detection, the culture medium was removed, and diluted 2′,7′-dichlorodihydrofluorescein diacetate (DCFH-DA) was added to the cells. The cells were incubated for 20 min at 37°C, then washed three times with serum-free culture medium to completely remove any unincorporated DCFH-DA. Cells were then washed with 1 mL of PBS by centrifugation at 1,500 rpm for 5 min, and the supernatant was discarded. Finally, ROS content was determined after resuspending the cells in 300 μL of PBS.

### 2.4 Immunofluorescence detection

Cells were washed three times with PBS for 3 min each and then fixed using 4% paraformaldehyde for 15 min. After washing, cells were permeabilized with 0.5% Triton X-100 in PBS at room temperature for 20 min. Glass slides were washed three times in PBS for 5 min each. Excess PBS was drained, and 5% BSA was added to block nonspecific binding at 37℃ for 30 min. The blocking solution was removed using a pipette, and primary antibody was added to each culture dish and incubated overnight at 4°C.

Following primary antibody incubation, the dishes were washed three times with PBS for 5 min each at 37°C. Diluted fluorescent secondary antibody was applied and the dishes were incubated again at 37°C for 30 min. After washing with PBS, DAPI stain was added and the dishes were incubated in the dark for 3 min to label the nuclei. Excess DAPI was removed before adding 50% glycerin to mount the cells. The culture dishes were examined under a fluorescence microscope, and images were captured for analysis.

### 2.5 ELISA and biochemical detection

Intracellular oxidative damage indicators like MDA, SOD, and rat interleukin 1 beta (IL-1β) levels were measured in accordance with the kit’s instructions.

### 2.6 Detection of quantitative polymerase chain reaction (qPCR)

Cells were lysed using Trizol reagent for total RNA extraction, with 0.2 mL of chloroform added per 1 mL of Trizol used. mRNA was isolated using an mRNA ultrapure purification kit. The concentration and purity of the extracted mRNA were assessed using an ultraviolet-visible spectrophotometer (NP80, NanoPhotometer), measuring the optical density at 260 and 280 nm (OD260/OD280). Complementary DNA was synthesized using an mRNA reverse transcription kit.

Quantitative real-time PCR was performed with a fluorescence PCR instrument. The thermal cycling conditions were as follows: initial pre-denaturation at 95°C for 10 min, followed by 40 cycles of denaturation at 95°C for 10 s, annealing at 58°C for 30 s, and elongation at 72°C for 30 s. B-actin served as an internal control. Gene expression levels were quantified using the 2^−ΔΔCt^ method. The sequences of the primers used are detailed in Table 1.

**Table 1:** Primer sequence

Primer name	Primer sequence (5′−3′)
GAPDH F	GACAACTTTGGCATCGTGGA
GAPDH R	ATGCAGGGATGATGTTCTGG
HCN1 F	GTCATCACCAAGTCCAGTAAAGAA
HCN1 R	GTAAAGGCGACAGTATGTATCAGC

### 2.7 WB detection

Total protein was extracted from the cells using RIPA buffer. After extraction, the lysate was centrifuged at a high speed of 12,000 rpm for 10 min at 4°C, and the precipitate was discarded. The supernatant was transferred into a new centrifuge tube. Protein concentration was then determined using a BCA protein assay kit.

For protein separation, the samples were denatured and subjected to sodium dodecyl sulfate–polyacrylamide gel electrophoresis for 1.5 h. This was followed by the electrotransfer of proteins onto a PVDF membrane at a constant current of 300 mA for another 1.5 h. The PVDF membrane was blocked using defat dried milk and incubated with the primary antibody overnight at 4°C.

The following day, the membrane was incubated with a secondary antibody at room temperature for 2 h, then exposed to ECL luminescent liquid. Subsequently, an ultrasensitive chemiluminescence imaging system was used to visualize the protein bands. The specific antibodies used and their corresponding dilution factors are listed in Table 2.

**Table 2:** Antibodies used and corresponding dilution factor

Antibody name	Dilution factor
ACTIN	1:2,000
IgG (H + L)	1:2,000
P65	1:1,000
IKKβ	1:1,000
IκB	1:1,000
p-IκB	1:1,000
NLRP3	1:1,000

### 2.8 Statistical analysis

All results are presented as mean ± standard deviation (*X* ± *S*). Statistical analyses were conducted using SPSS 20.0 software. Differences among multiple groups were evaluated using one-way ANOVA, with a significance level set at *α* = 0.05; thus, a *P*-value <0.05 indicates a statistically significant difference. Graphs were generated using GraphPad Prism 9.0 software. The grayscale value analysis of WB bands was performed using ImageJ software.

## 3 Experimental results

### 3.1 HCN1 overexpression (HCN1-OE) significantly increases HCN1 expression in H9C2 cells

**Figure 1:**
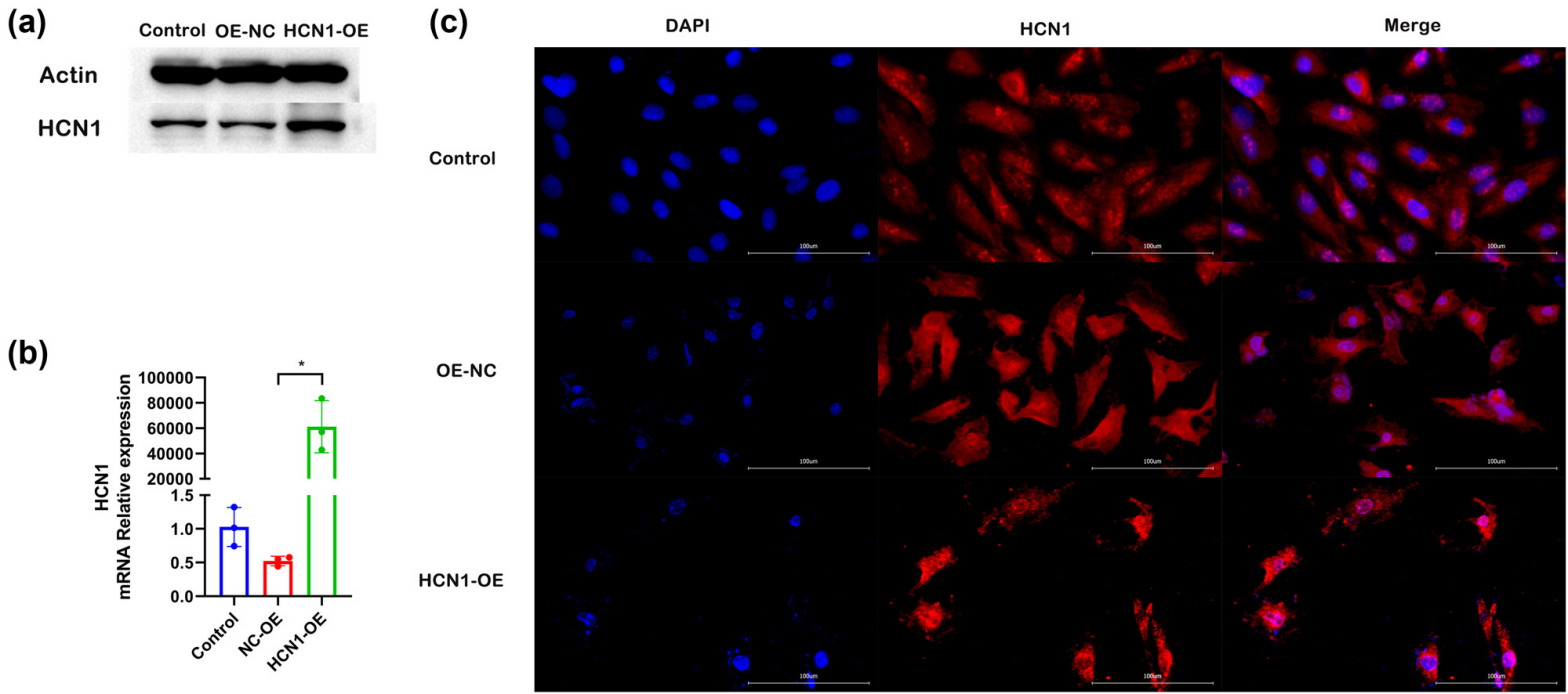
HCN1 expression level after HCN1 overexpression transfection in H9C2 cells ((a) WB, (b) PCR, and (c) immunofluorescence; *P < 0.05; *n* = 3).

To examine the impact of HCN1-OE on the expression levels of HCN1 in rat cardiomyocytes (H9C2), the expression of the HCN1 gene was first assessed in H9C2 cells following overexpression. The results of WB and PCR are displayed in Figure 1a and b. Cells transfected with the HCN1-OE construct exhibited substantially higher levels of HCN1 compared to those in the normal and overexpression control groups, with statistically significant differences observed. Immunofluorescence data (Figure 1c) confirmed these findings, showing a marked increase in fluorescence intensity post-transfection, indicative of elevated HCN1 expression.

### 3.2 Effects of HCN1 overexpression on cell vitality

**Figure 2:**
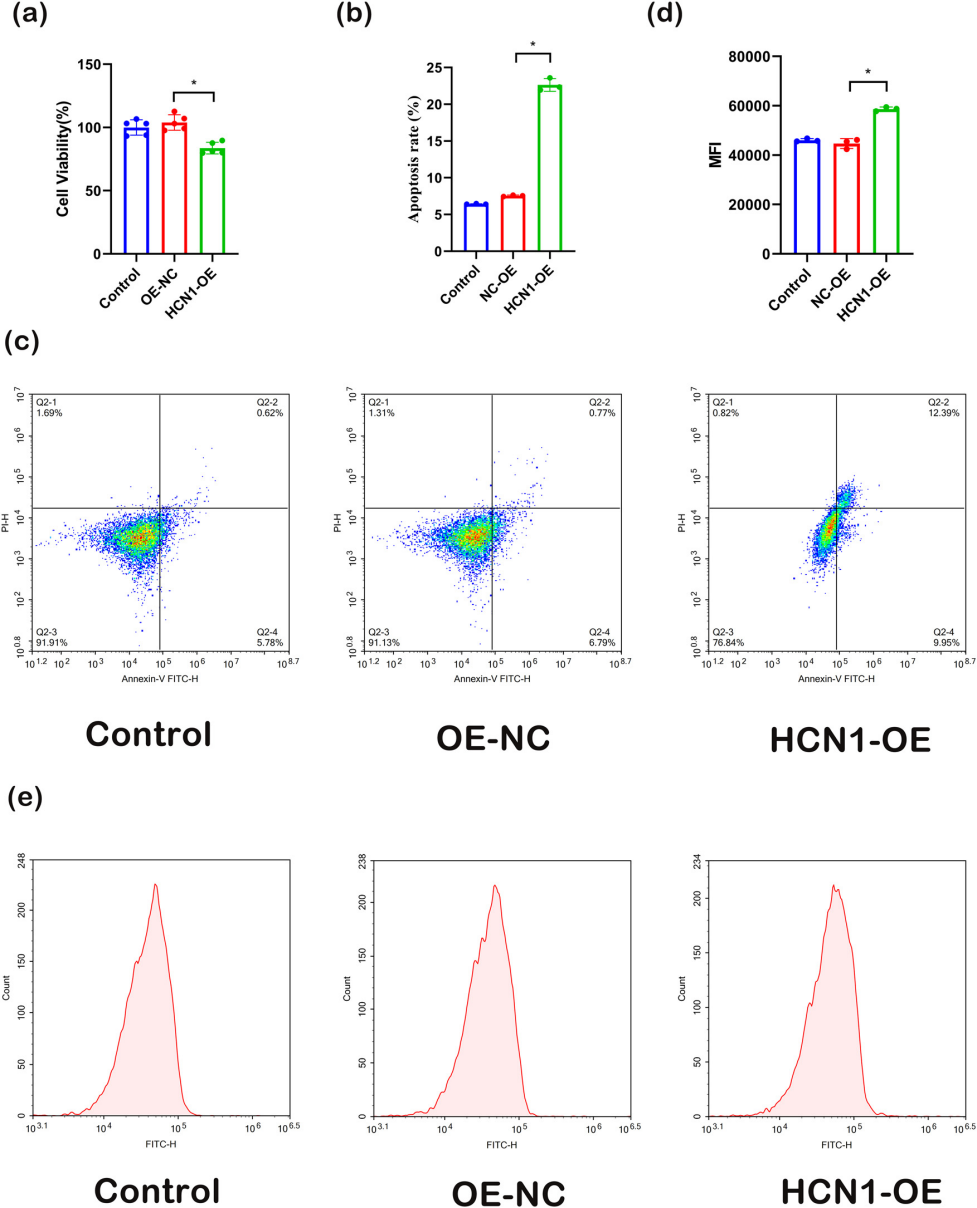
Effect of HCN1 overexpression on cell vitality ((a) CCK8 detection on cell vitality in different groups, (b) and (c) flow cytometry detection on cell apoptosis rate, (d) and (e) flow cytometry detection on cell ROS content in each group; *P < 0.05; *n* = 3).

To explore the effects of HCN1-OE on the viability of H9C2 cells, cell vitality was assessed using the CCK8 assay following transfection. The results (Figure 2a) indicate a significant reduction in cell vitality post HCN1-OE transfection. Additionally, flow cytometry was employed to analyze apoptosis (Figure 2b and c); there was a notable increase in cell apoptosis following HCN1-OE transfection. Furthermore, the results from flow cytometry-based ROS detection (Figure 2d and e) reveal a significant elevation in intracellular ROS levels after HCN1-OE transfection. Collectively, these outcomes demonstrate that HCN1-OE leads to an increase in cell apoptosis.

### 3.3 HCN1 overexpression inhibits the antioxidant power of H9C2 cells

**Figure 3:**
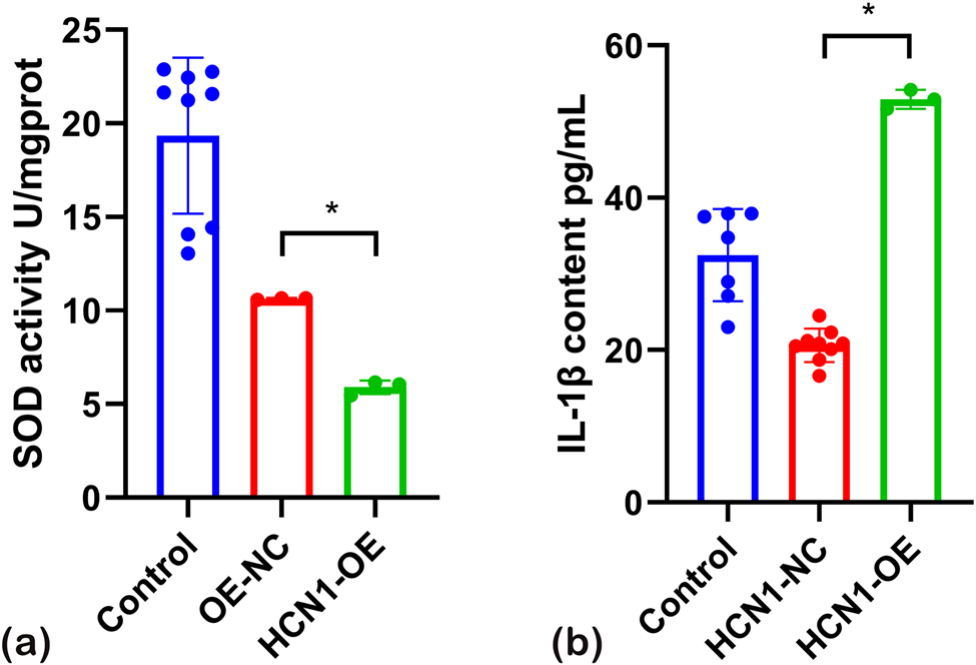
(a) SOD and (b) IL-1β results 6 utilizing ELISA (*P < 0.05; *n* = 3).

To assess the impact of HCN1-OE on the antioxidant capabilities of H9C2 cells, biochemical assays for SOD and rat IL-1β were conducted. The findings (Figure 3) show that SOD levels were significantly reduced in the HCN1-OE group compared to both the normal and overexpression control groups. Conversely, the levels of IL-1β were significantly elevated in the HCN1-OE group relative to the controls. These results suggest that overexpression of HCN1 not only increases cellular inflammation but also diminishes the antioxidant capacity of the cells.

### 3.4 Effects of NF-κB inhibitor PDTC on cells

**Figure 4:**
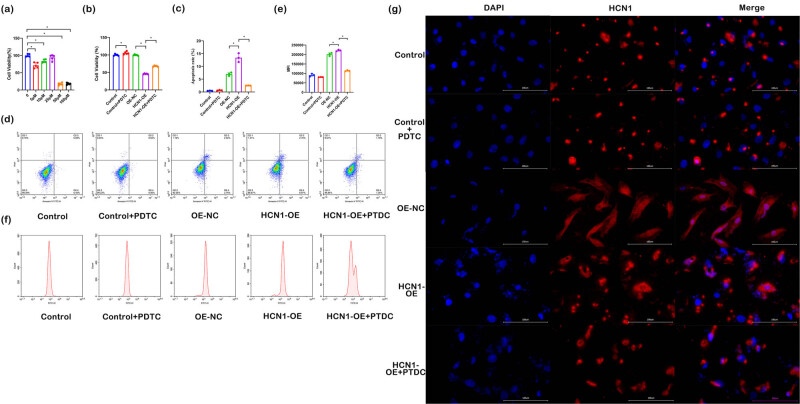
Effect of NF-κB inhibitor on cells ((a) CCK8 detection on H9C2 cells treated by PDTC with different concentrations; (b) CCK8 detection on cell vitality in different groups; (c) and (d) flow cytometry detection on cell apoptosis rate; (e) and (f) flow cytometry detection on cell ROS content in each group; (g) immunofluorescence detection on HCN1 protein expression in each group). ^*^ *P* < 0.05; *n* = 3).

To explore the effects of HCN1-OE on H9C2 cells and the potential modulatory role of the NF-κB inhibitor PDTC, the cells were treated with PDTC. Cell viability was assessed using the CCK8 assay (Figure 4a). After treating the cells with various concentrations of PDTC for 48 h, the optimal cell vitality was observed at a concentration of 25 μM, which was chosen for further experimental studies.

Subsequently, H9C2 cells were subjected to combined treatment with NF-κB inhibitor PDTC and HCN1-OE transfection for 48 h. CCK8 results (Figure 4b) indicate that cell vitality in the HCN1-OE group was decreased compared to the control group. However, cell vitality in the HCN1-OE + PDTC group was enhanced relative to the HCN1-OE group alone. Based on flow cytometry (Figure 4c and d), there is a significant increase in the apoptosis rate in the HCN1-OE group compared to the control, whereas apoptosis rates significantly decreased in the HCN1-OE + PDTC group compared to the HCN1-OE group.

Additionally, ROS levels measured by flow cytometry (Figure 4e and f) indicated a significant increase in ROS content in the HCN1-OE group compared to the control group. In contrast, ROS levels in the HCN1-OE + PDTC group decreased significantly compared to the HCN1-OE group. Immunofluorescence analysis (Figure 4g) revealed that HCN1 expression levels were lower in the HCN1-OE + PDTC group compared to the HCN1-OE group.

These findings suggest that PDTC treatment enhances cell survival following HCN1-OE.

### 3.5 Effects of NF-κB inhibitor PDTC on the antioxidant power of the cells

**Figure 5:**
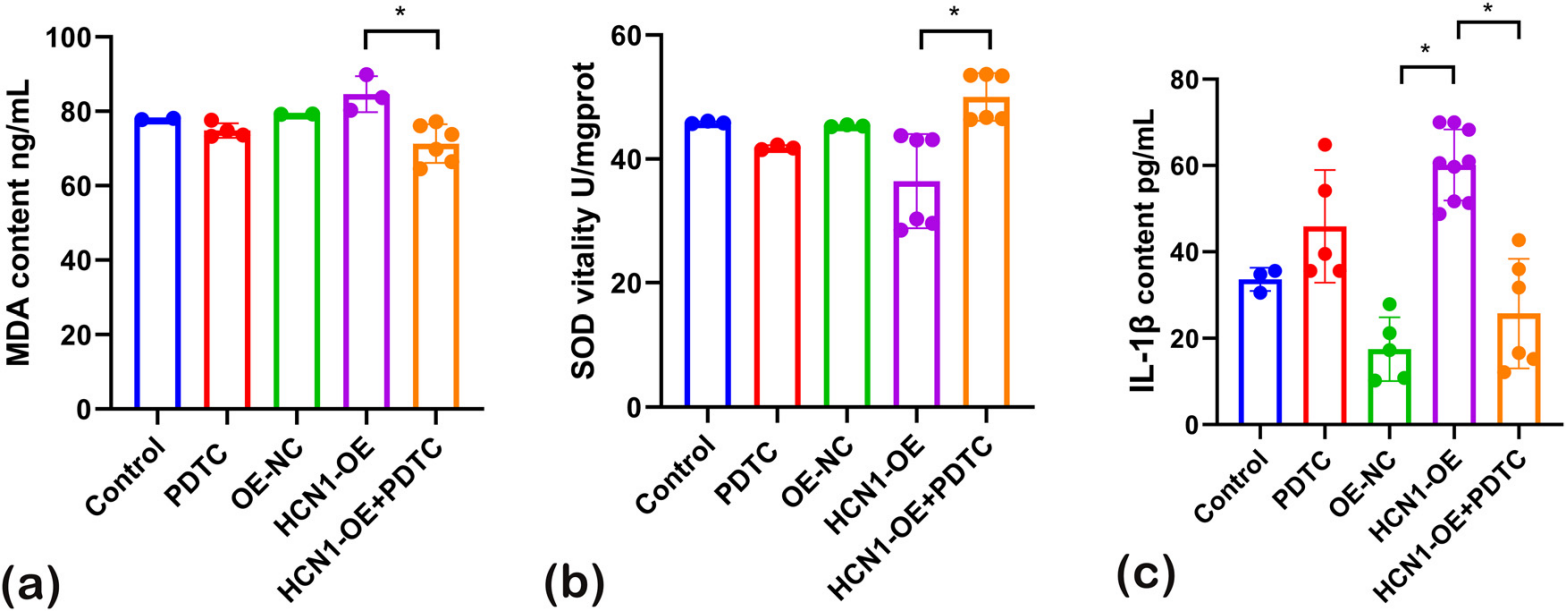
(a) MDA, (b) SOD and (c) IL-1β results utilizing ELISA (*P < 0.05; *n* = 3).

To assess the effects of the NF-κB inhibitor PDTC on cells transfected with HCN1-OE, biochemical assays were performed to measure intracellular oxidative damage markers, including MDA, SOD, and rat IL-1β (Figure 5).

In the HCN1-OE group, the level of MDA, a marker of lipid peroxidation, was elevated compared to the normal group, indicating increased oxidative stress. However, MDA levels significantly decreased in the HCN1-OE + PDTC group compared to the HCN1-OE group. Conversely, SOD levels were reduced in the HCN1-OE group compared to the normal group. Treatment with PDTC resulted in a significant increase in SOD levels in the HCN1-OE + PDTC group compared to the HCN1-OE group.

Furthermore, IL-1β levels were lower in the HNC1-OE group than in the normal group. However, in the HCN1-OE + PDTC group, IL-1β levels were significantly higher than in the HCN1-OE group, suggesting that PDTC may help mitigate the inflammatory impact of HCN1-OE.

### 3.6 Effects of HCN1-OE on NF-κB signaling pathway

**Figure 6:**
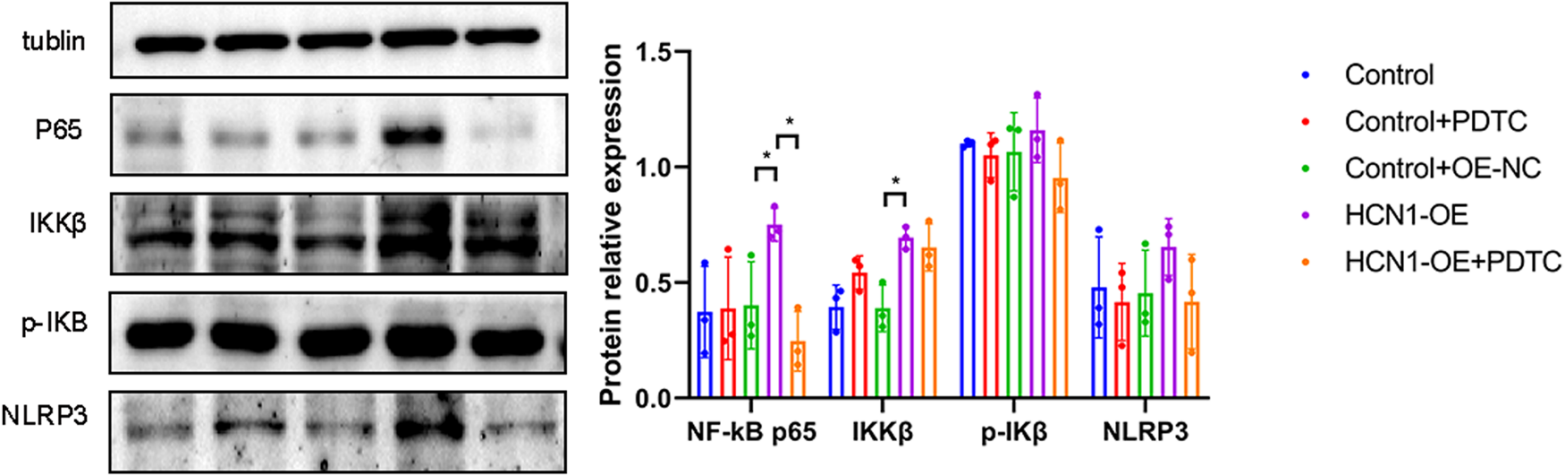
WB analysis of the impact of NF-κB inhibitor on NF-κB pathway (*P < 0.05; *n* = 3).

To assess the effects of NF-κB inhibitors on cells with HCN1-OE, WB was utilized to measure the expression of relevant proteins (Figure 6). After the overexpression transfection, significant alterations were observed in the expression of several key proteins involved in the NF-κB signaling pathway.

NF-κB p65: There was a significant increase in the HCN1-OE group compared to the control and OE-NC groups. Notably, NF-κB p65 levels significantly decreased in the HCN1-OE + PDTC group compared to the HCN1-OE group.

IKKβ: Similarly, IKKβ levels were significantly elevated in the HCN1-OE group in comparison to the control and OE-NC groups. IKKβ levels were significantly lower in the HCN1-OE + PDTC group than in the HCN1-OE group.

p-IκB: The phosphorylated form of IκB was higher in the HCN1-OE group than in the control and OE-NC groups, indicating increased NF-κB activation. However, p-IκB levels were reduced in the HCN1-OE + PDTC group compared to the HCN1-OE group.

Nucleotide-binding domain and leucine rich repeat containing protein (NLRP3): There was an increase in NLRP3 levels in the HCN1-OE group compared to the control and OE-NC groups, with a subsequent decrease observed in the HCN1-OE + PDTC group compared to the HCN1-OE group.

## 4 Discussion

AMI is a critical disorder that poses significant risks to human health. A key pathological event following myocardial infarction involves the inflammatory response and cardiomyocyte apoptosis, which are mediated by the IKKβ/NF-κB signaling pathway. NF-κB is a multifunctional transcriptional protein in cardiomyocytes, with NF-κB p65 being its predominantly active form. Under physiological conditions, NF-κB and NF-κB p65 exist as inactive trimers in the cytoplasm [16]. Upon the onset of AMI, stimuli activate membrane receptors leading to the recruitment and activation of the cytoplasmic IKK complex. This complex phosphorylates the N-terminus of NF-κB at Ser32 and Ser36, resulting in the dissociation of the NF-κB/NF-κB p65 trimer and the release of NF-κB p65 [17]. The liberated NF-κB p65 then translocates into the nucleus, where it mediates the transcription of various inflammation-related genes. Concurrently, the elevated nuclear expression of pro-inflammatory genes further enhances the synthesis and secretion of inflammatory mediators such as IL-1β, TNF-α, and IFN-γ, leading to myocardial tissue damage and creating a detrimental cycle of inflammation and transcription [18].

The WB results in this study show significant increases in NF-κB p65, IKKβ, and p-IκB levels following HCN1-OE transfection. Additionally, flow cytometry results indicate a marked decrease in cell viability post-transfection, corroborating that HCN1-OE activates the NF-κB pathway, confirming its role in exacerbating inflammatory responses post-AMI.

According to previous studies, NF-κB is highly activated in various cardiovascular diseases and plays a significant role in exacerbating myocardial damage [19]. This activation is particularly evident in conditions such as myocardial ischemia/reperfusion injury and post-myocardial infarction heart remodeling [20]. NF-κB mediates detrimental processes such as autophagy in these settings. In heart failure models, the administration of PDTC, an NF-κB inhibitor, has been shown to ameliorate cardiac dysfunction and significantly reduce cardiomyocyte apoptosis [21]. These findings underscore the pivotal role of NF-κB in the pathophysiology of cardiovascular diseases, suggesting that targeting NF-κB could be a strategic therapeutic approach. By mitigating the inflammatory and autophagic processes mediated by NF-κB, it may be possible to alter the course of disease progression and improve clinical outcomes for patients suffering from myocardial infarction and heart failure.

Additionally, PDTC has demonstrated protective effects in diabetic cardiomyopathy by reducing the synthesis of inducible nitric oxide synthase and nitrotyrosine in myocardial tissues [22]. It also improves myocardial contractility in cirrhotic cardiomyopathy by preventing the degradation of I-κB, thereby inhibiting NF-κB activation [23].

The results of this study reveal that introduction of PDTC following HCN1-OE transfection resulted in increased cell vitality and a significant reduction in the levels of key inflammatory mediators such as NF-κB p65, IKKβ, p-IκB, compared to the HCN1-OE only group.

The NLRP3 inflammasome is a macromolecule multiprotein complex that consists of NLRP3, apoptosis-associated speck-like protein (ASC) and Caspase-1 proteins. Overactivation of the NLRP3 inflammasome can lead to the cleavage of IL-1β precursor and IL-18 precursor into mature IL-1β and IL-18 through activated Caspase-1. These cytokines activate downstream signaling pathways, triggering the production of numerous inflammatory mediators, and leading to severe inflammatory responses and subsequent tissue damage. Current research highlights the significant role of the NLRP3 inflammasome pathway in the pathogenesis of atherosclerotic conditions such as myocardial infarction and ischemic cerebral infarction, positioning it as a potential therapeutic target in atherosclerosis management. This area of investigation opens new avenues for therapeutic interventions, emphasizing the critical need to understand the complex molecular and cellular mechanisms that underpin these cardiovascular events. Elucidating the role of the NLRP3 inflammasome in atherosclerosis can pave the way for the development of novel pharmacological agents aimed at modulating this pathway, thereby potentially mitigating the adverse outcomes associated with myocardial infarction and ischemic cerebral infarction [24].

WB results in this study demonstrated a significant increase in NLRP3 levels in the HCN1-OE group compared to the control group. Additionally, biochemical assays revealed elevated levels of MDA and IL-1β in the HCN1-OE group, indicative of increased oxidative stress and inflammation, respectively. Upon administration of PDTC, a notable reduction in NLRP3 levels was observed via WB, and levels of MDA and IL-1β also decreased significantly compared to the HCN1-OE only group.

## 5 Conclusion

The increased expression of HCN1 significantly activates the NF-κB signaling pathway, presenting substantial implications for further research. Following transfection with HCN1-OE, a notable reduction in cell vitality was observed. The introduction of PDTC markedly improved cell vitality compared to the HCN1-OE group alone. WB analyses revealed significant elevations in the levels of NF-κB p65, IKKβ, p-IκB, NLRP3, Beclin-1, and LC3 II/I in the HCN1-OE group compared to the control group. However, these levels significantly decreased following the PDTC treatment. While the exact mechanisms through which PDTC modulates these processes remain unclear, this study suggests promising avenues for further research into the prevention and treatment of myocardial infarction.
